# Longitudinal mitochondrial bioenergetic signatures of blood monocytes and lymphocytes improve during treatment of drug-susceptible pulmonary tuberculosis patients

**DOI:** 10.3389/fimmu.2024.1465448

**Published:** 2024-11-13

**Authors:** Bridgette M. Cumming, Kelvin W. Addicott, Fernanda Maruri, Vanessa Pillay, Rukaya Asmal, Sashen Moodley, Beatriz Barreto-Durate, Mariana Araújo-Pereira, Matilda Mazibuko, Zoey Mhlane, Nikiwe Mbatha, Khadija Khan, Senamile Makhari, Farina Karim, Lauren Peetluk, Alexander S. Pym, Mahomed Yunus S. Moosa, Yuri F. van der Heijden, Timothy S. Sterling, Bruno B. Andrade, Alasdair Leslie, Adrie J. C. Steyn

**Affiliations:** ^1^ Africa Health Research Institute, University of KwaZulu-Natal, Durban, South Africa; ^2^ Vanderbilt Tuberculosis Center, Vanderbilt University School of Medicine, Nashville, TN, United States; ^3^ Division of Infectious Diseases, Department of Medicine, Vanderbilt University School of Medicine, Nashville, TN, United States; ^4^ Multinational Organization Network Sponsoring Translational and Epidemiological Research (MONSTER) Initiative, Salvador, Brazil; ^5^ Laboratório de Pesquisa Clínica e Translacional, Instituto Gonçalo Moniz, Fundação Oswaldo Cruz, Salvador, Bahia, Brazil; ^6^ Department of Infectious Diseases, University of KwaZulu-Natal, Durban, South Africa; ^7^ Global Division, The Aurum Institute, Johannesburg, South Africa; ^8^ Department of Infection and Immunity, University College of London, London, United Kingdom; ^9^ Department of Microbiology, University of Alabama at Birmingham, Birmingham, AL, United States; ^10^ Centers for AIDS Research and Free Radical Biology, University of Alabama at Birmingham, Birmingham, AL, United States

**Keywords:** tuberculosis, bioenergetic metabolism, lymphocytes, monocytes, TB treatment, cytokines, Seahorse XF96

## Abstract

The impact of human pulmonary tuberculosis (TB) on the bioenergetic metabolism of circulating immune cells remains elusive, as does the resolution of these effects with TB treatment. In this study, the rates of oxidative phosphorylation (OXPHOS) and glycolysis in circulating lymphocytes and monocytes of patients with drug-susceptible TB at diagnosis, 2 months, and 6 months during treatment, and 12 months after diagnosis were investigated using extracellular flux analysis. At diagnosis, the bioenergetic parameters of both blood lymphocytes and monocytes of TB patients were severely impaired in comparison to non-TB and non-HIV-infected controls. However, most bioenergetic parameters were not affected by HIV status or glycemic index. Treatment of TB patients restored the % spare respiratory capacity (%SRC) of the circulating lymphocytes to that observed in non-TB and non-HIV infected controls by 12 months. Treatment also improved the maximal respiration of circulating lymphocytes and the %SRC of circulating monocytes of the TB patients. Notably, the differential correlation of the clinical and bioenergetic parameters of the monocytes and lymphocytes from the controls and TB patients at baseline and month 12 was consistent with improved metabolic health and resolution of inflammation following successful TB treatment. Network analysis of the bioenergetic parameters of circulating immune cells with serum cytokine levels indicated a highly coordinated immune response at month 6. These findings underscore the importance of metabolic health in combating TB, supporting the need for further investigation of the bioenergetic immunometabolism associated with TB infection for novel therapeutic approaches aimed at bolstering cellular energetics to enhance immune responses and expedite recovery in TB patients.

## Introduction

Bioenergetics has become central to understanding the pathology of human noncommunicable diseases such as neurodegeneration, diabetes, cancer, and cardiovascular diseases (1–6). Consequently, bioenergetics has also been extensively examined to discover novel therapeutic strategies for a wide range of diseases (7). Increasing evidence suggests that systemic bioenergetic capacity is reflected in blood-based mitochondrial respirometric profiling. For example, mitochondrial respiratory parameters measured in peripheral blood mononuclear cells (PBMC) have been linked to various age-related diseases and disorders, such as Alzheimer’s disease, diabetes, fibromyalgia, septicemia, and ischemic and valvular heart disease (8–14).

Furthermore, it is now well established that the metabolic reprogramming of immune cells is a central step in directing immune function and cytokine and chemokine production (7). Therefore, bioenergetic metabolism is likely to play an important role in the progression of infectious diseases. Infection of human monocyte-derived macrophages with *Mycobacterium tuberculosis* (*Mtb*) *in vitro* has been shown to decelerate glycolysis and alter mitochondrial substrate dependency from glucose to fatty acids (15, 16). It has been demonstrated that CD4+ and CD8+ T cells and NK cells from HIV-positive, treatment-naïve patients have reduced basal and maximal respiration compared with HIV-negative individuals (17). Anti-viral treatment restored the bioenergetic phenotype of CD8+ T cells and NK cells, but respiratory impairment persisted in CD4+ T cells. Examination of host bioenergetic metabolism in response to *Mtb* infection has been conducted both *in vitro* and in animal models (15, 16, 18). However, there remains a gap in our understanding of how human pulmonary TB influences the bioenergetic metabolism of immune cells in the peripheral circulation, and how these alterations resolve with drug treatment. As the bioenergetic metabolism of immune cells in TB patients remains unclear, it presents a promising opportunity for developing new therapeutic interventions, improving diagnostic and prognostic tools, and deepening our understanding of the pathophysiology of TB.

**Figure 1:**
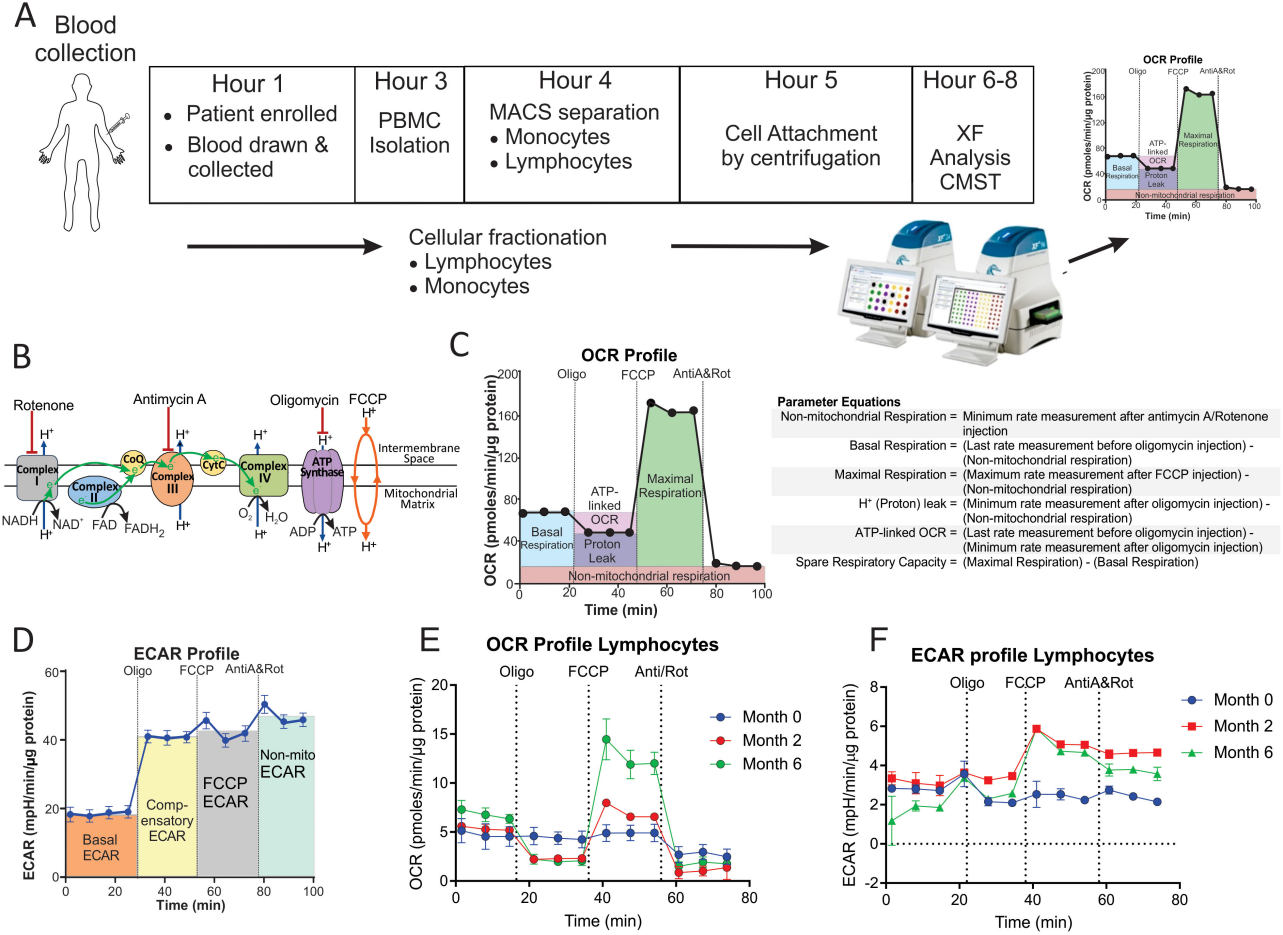
Measurement of the bioenergetic parameters from the Cell Mito Stress Test profiles. **(A)** Flow diagram of the workflow for the processing of each blood sample. **(B)** Schematic representation of the electron transport chain and OXPHOS demonstrating targets of the compounds used in the Cell Mito Stress Test (CMST). **(C)** OCR profile showing measurement of respiratory bioenergetic parameters. **(D)** ECAR profile demonstrating measurement of ECAR bioenergetic parameters. **(E, F)** Representative **(E)** OCR profile and **(F)** ECAR profiles of a TB patient at baseline (Month 0), Month 2 and Month 6 following initiation of treatment. Anti-A, antimycin-A; FCCP, carbonyl cyanide-4-(trifluoromethoxy)phenylhydrazone; MACS, magnetic activated cell sorting; Non-mito, non-mitochondrial; Oligo, oligomycin; PBMC, peripheral blood mononuclear cells; Rot, rotenone; XF, extracellular flux.

The bioenergetic metabolism of immune cells can be examined noninvasively in real time using extracellular flux analysis with Agilent Seahorse XF technology. Extracellular flux analysis measures changes in both the oxygen consumption rate (OCR) and the extracellular acidification rate (ECAR) of cells. OCR and ECAR are proportional to the rates of mitochondrial oxidative phosphorylation (OXPHOS) and glycolysis in the cytoplasm, respectively. Using a Cell Mito Stress Test (CMST) (**Figure 1A**), OCR and ECAR measurements were used to determine 12 bioenergetic parameters that indicate mitochondrial and glycolytic function in the cells (**Figures 1B–D**). To understand the role of the bioenergetic metabolism of immune cells in TB, extracellular flux analysis has been used to investigate the bioenergetic metabolism of human and mouse-derived macrophages infected *in vitro* with *M. tuberculosis (Mtb)* (15, 18), in addition to the alteration of CD8^+^ T cell metabolism during chronic TB infection in mice (16). Infection with live *Mtb* was found to suppress both mitochondrial and glycolytic functions in human macrophages (15), while chronic TB infection suppressed mitochondrial respiration of CD8+ T cells in mice (16).

In this study, we hypothesized that *Mtb* infection induces bioenergetic impairment in circulating immune cells, leading to reduced metabolic activity and functionality. We also posit that anti-TB drug therapy will restore the bioenergetic capacity of these immune cells, reversing their impairment and enhancing their ability to mount an effective immune response against infection. To test this hypothesis, we examined the bioenergetic metabolism of monocytes and lymphocytes in the whole blood of drug-susceptible TB patients undergoing treatment compared with euglycemic and HIV-negative controls recruited from the same district. We also evaluated the correlation between the bioenergetic parameters and serum cytokine levels in response to anti-TB treatment. By adapting a real-time, noninvasive bioenergetic platform to study the bioenergetics of immune cells from TB patients, we have generated new knowledge that may help elucidate how bioenergetic changes impact immunity, which may lead towards new immunomodulatory strategies and identify individuals at risk of disease progression or relapse.

## Results

### Baseline characteristics of the study population

The characteristics of the study population and the bioenergetic parameters measured at baseline are shown in **Table 1**. Thirty-nine GeneXpert-positive, solid culture-positive drug-susceptible participants were included in the study.

**Table 1:** Baseline clinical and demographic characteristics of culture positive, drug susceptible TB cases and healthy controls.

Bioenergetics Data N = 71 n (%)			
Characteristic N (%) or Median [IQR]	Culture Positive (n = 39)	TB uninfected individuals (n = 32)	P-value^a^
Age, median (IQR), years	36 [26, 46]	23 [21, 31]	0.0001
Male sex	28 (72)	14 (44)	0.0168
HIV positive	22 (56)	0 (0)	<0.0001
Currently on ARVs	8/22 (36)	0 (0)	
HbA1c, median (IQR), %	5.90 [5.40, 6.20]	5.40 [5.15, 5.50]	<.0001
HbA1c <5.7%	12 (31)	28 (88)	<0.0001
HbA1c 5.7 to 6.4%	23 (59)	4 (12)	
HbA1c ≥6.5%	4 (10)	0 (0)	

a Abs, absolute count; BMI, body mass index; HbA1c, Hemoglobin A1c (glycated); IQR, interquartile range; MGIT, Mycobacterial Growth Indicator Tube.

### TB impairs the bioenergetic metabolism of circulating monocytes and lymphocytes

**Figure 2:**
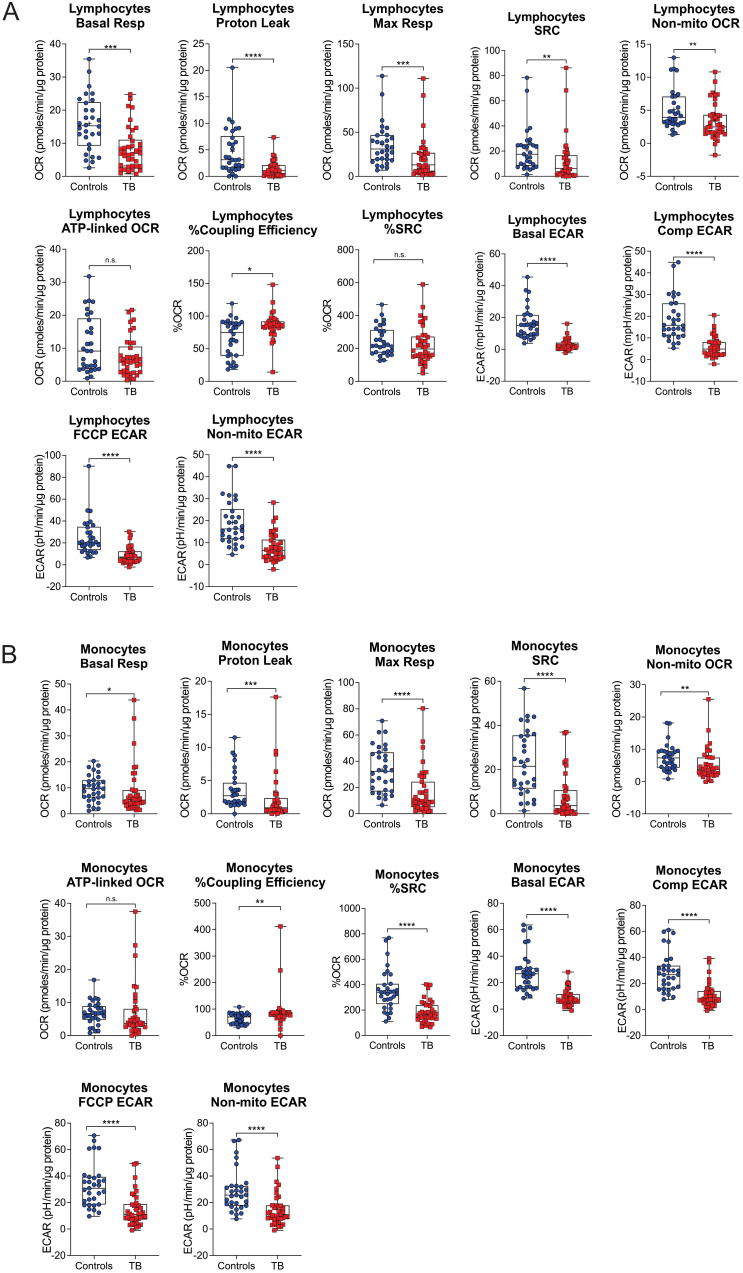
Baseline measurements of the bioenergetic parameters of the **(A)** lymphocytes and **(B)** monocytes of TB patients (n = 39) in comparison to those of asymptomatic HIV negative individuals (n = 32). TB patients and healthy controls were recruited from the same study site. Significant differences are observed in most of the bioenergetic parameters of both the monocytes and lymphocytes, with a few exceptions: %Spare Respiratory Capacity (%SRC) in the lymphocytes, and ATP*-*linked OCR in the monocytes. Comp, compensatory; Max, maximum; Non-mito, non-mitochondrial; Resp, respiration. *P<0.05; **P<0.01; ***P<0.001; ****P<0.0001; ns, not significant.

**Figure 3:**
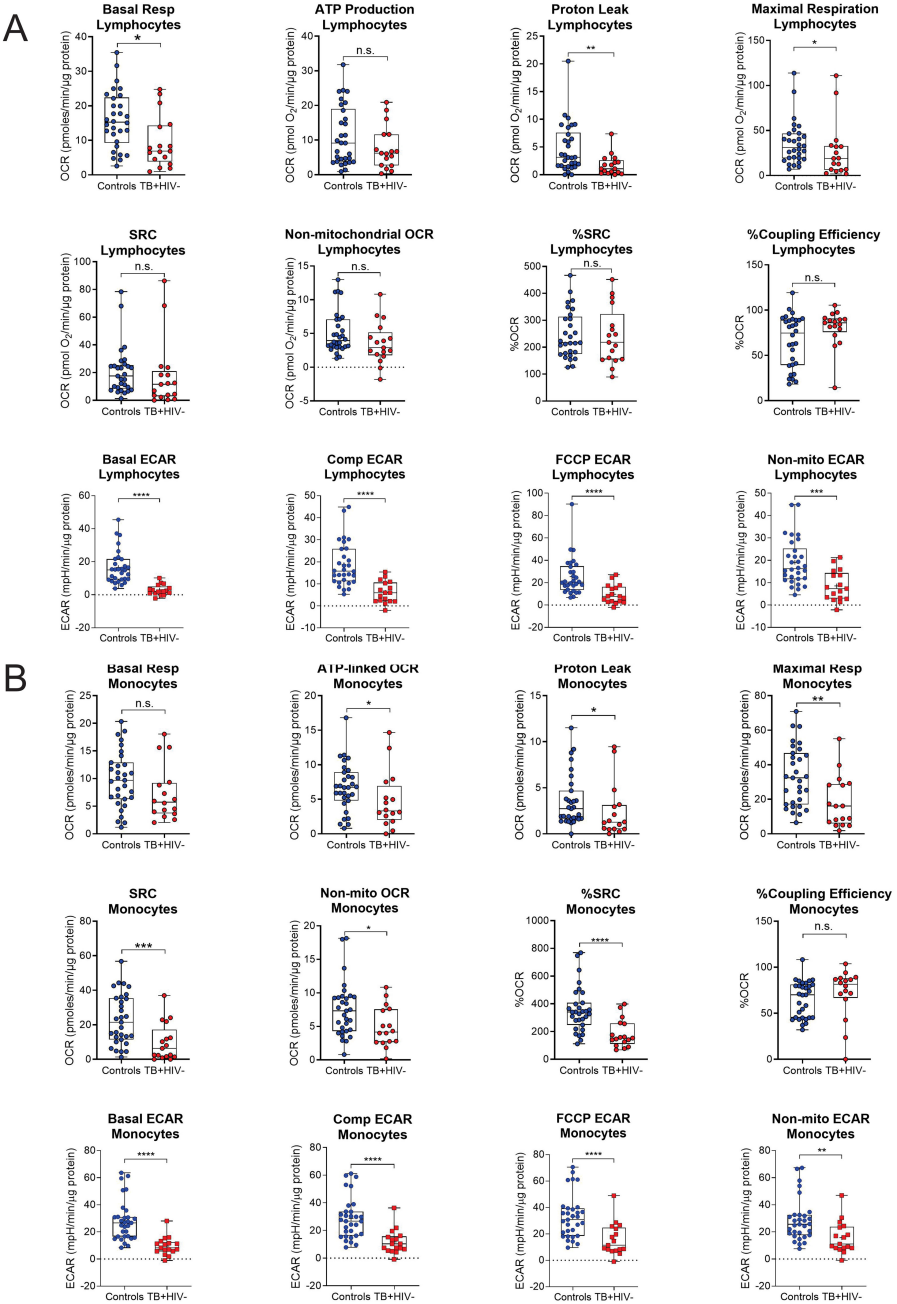
Baseline measurements of the bioenergetic parameters of the **(A)** lymphocytes and **(B)** monocytes of the asymptomatic HIV negative individuals (n = 30) and TB HIV-negative patients (n = 17). TB HIV-negative patients and asymptomatic HIV-negative individuals were recruited from the same study site. In the lymphocytes, significant differences are observed in basal respiration, proton leak, maximal respiration and ECAR parameters. In the monocytes, significant differences were observed in most of the bioenergetic parameters of both the monocytes, except for basal respiration and %Coupling Efficiency. Comp, compensatory; Max, maximum; Non-mito, non-mitochondrial; Resp, respiration. *P<0.05; **P<0.01; ***P<0.001; ****P<0.0001: ns, not significant.

The bioenergetic status of the monocytes and lymphocytes isolated from the blood of the participants was determined using extracellular flux analysis within 8 h of collection (**Figure 1A**). Lymphocytes are isolated by negative selection and are therefore comprised of a mixed population of CD4+ and CD8+ T cells, together with B cells, NK cells, dendritic cells, and neutrophils. Ten bioenergetic parameters were calculated from the OCR and ECAR profiles generated by the cell mito stress test (**Figures 1B–D**). Representative OCR and ECAR profiles of a TB patient are shown at baseline (Month 0), Month 2, and Month 6 following the initiation of treatment (**Figures 1E, F**). At baseline, the bioenergetic parameters of the circulating lymphocytes and monocytes from TB patients with or without HIV co-infection were severely impaired compared to those of the controls, except for ATP-linked OCR, which was not significantly different for either cell subset (**Figures 2A, B**). Among the TB patients, apart from the non-mitochondrial OCR of the lymphocytes, the bioenergetic parameters of those with HIV co-infection did not differ significantly from those of HIV-negative TB patients (**Supplementary Figures S1A, B**), implying that bioenergetic differences between TB participants and controls may not be driven by HIV. However, significant bioenergetic differences were observed between HIV-negative TB patients and asymptomatic HIV-negative controls (**Figures 3A, B** **),** suggesting that TB infection may have a greater influence on the bioenergetics of circulating monocytes and lymphocytes in this cohort. The bioenergetic parameters of both monocytes and lymphocytes did not appear to be influenced by the glycemic status/index of the TB patients, as no significant differences were observed in the parameters of the monocytes and lymphocytes among TB patients with HbA1c levels <5.7%, 5.7%–6.4% or ≥6.5% (**Supplementary Figures S2A, B**). However, significant bioenergetic differences were observed between the HbA1c level groups of TB patients and asymptomatic HIV-negative controls (**Supplementary Figures S2A, B**), alluding to the impact of TB infection on the bioenergetics of circulating cells.

In summary, the significant difference in the bioenergetic parameters between healthy controls and TB patients at diagnosis underscores the important role of host bioenergetic metabolism in the immune response to microbial disease. In comparison to healthy controls, the decreased values observed in the bioenergetic parameters of monocytes and lymphocytes suggest a compromised metabolic capacity within immune cells among TB patients, hindering their ability to mount an effective response against TB.

### %SRC of both lymphocytes and monocytes increased on completion of treatment

To examine any changes in bioenergetic metabolism during and after treatment, the bioenergetic parameters of circulating monocytes and lymphocytes of the TB patients were examined at months 2 and 6 during TB treatment and at month 12 following diagnosis. The number of participants with follow-up visits analyzed at months 2 (n = 23) and 6 (n = 25) was reduced due to the multifaceted nature of our experimental design, which permitted the analysis of only one participant per day. Consequently, data for all four time points [baseline (M0), month 2 (M2), month 6 (M6), and month 12 (M12)] were collected for the nine TB cases.

**Figure 4:**
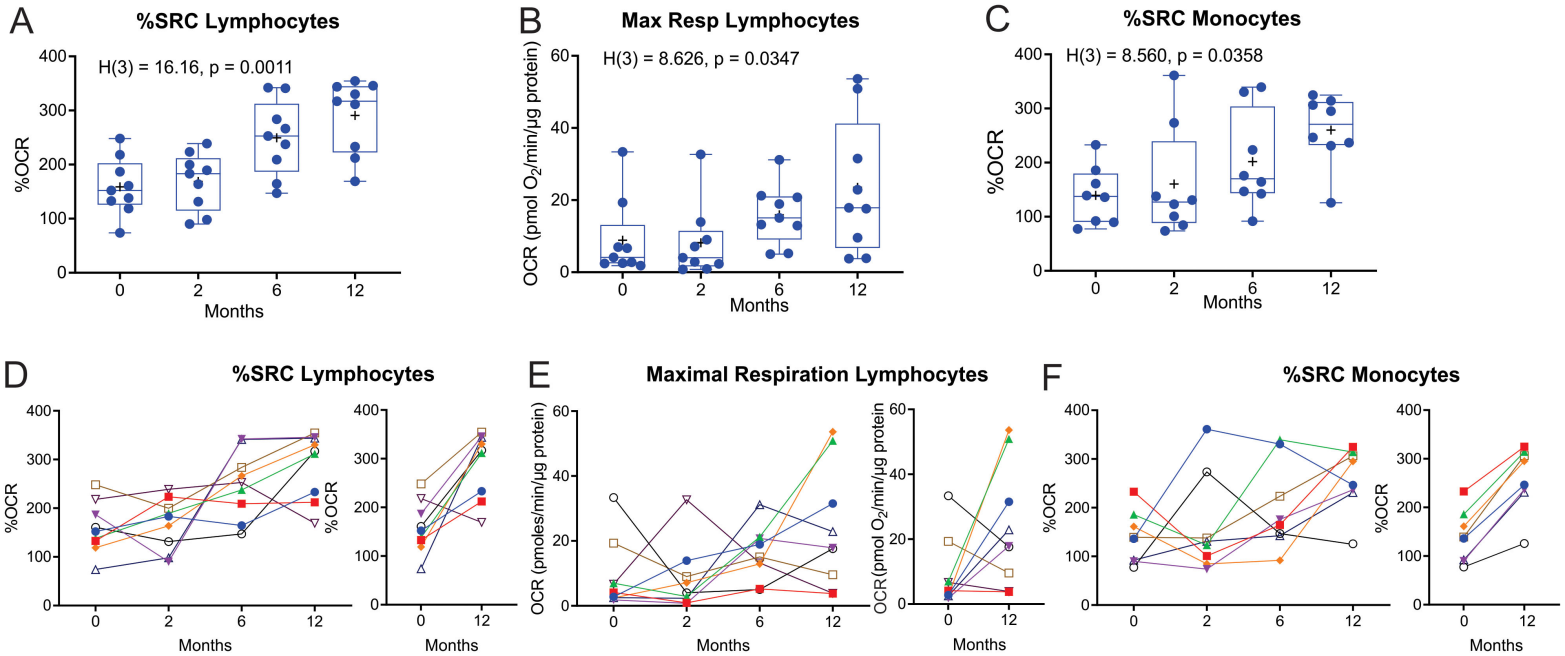
Longitudinal measurement of the %SRC and maximal respiration of circulating immune cells of nine TB patients with data at all four timepoints during and after TB treatment. **(A–C)** Changes in %SRC **(A)** and Max Resp **(B)** of the circulating lymphocytes, and **(C)** %SRC of the monocytes of nine TB patients during and after treatment. Dotted line represents the median of non-TB infected, HIV-negative control levels. **(D–F)** Individual line plots of the %SRC **(D)** and Max Resp **(E)** of the circulating lymphocytes, and **(F)** %SRC of the monocytes, of each of the nine TB patients during and after treatment; individual comparisons of the %SRC at diagnosis (0 months) and 12 months (6 months after completion of TB treatment) are also shown. KW, Kruskal–Wallis statistic.

Significant longitudinal changes were observed in both the median and mean %Spare Respiratory Capacity (%SRC, Kruskal–Wallis statistic = 16.16, *P* = 0.0011) and maximal respiration (Max Resp, Kruskal–Wallis statistic = 8.63, *P* = 0.0347) of the lymphocytes (**Figures 4A, B**). SRC is an indicator of a cell’s ability to increase OCR under conditions of stress. Line plots of %SRC of the lymphocytes of the individual cases illustrate incremental increases in the %SRC with distinct increases observed in eight of the nine patients with TB between M0 and M12 (**Figure 4D**). The Max Resp of lymphocytes was increased in five of the nine patients with TB by M12 (**Figure 4E**).

The %SRC of monocytes also changed longitudinally (Kruskal-Wallis statistic = 8.560, *P* = 0.0358) in eight of the TB participants that had data at all four time points (**Figure 4C**). Line plots of the %SRC of monocytes from the individual cases indicate substantial improvement between M0 and M12, with an overall increase in %SRC by M12 (**Figure 4F**). The initial low %SRC values in the monocytes at baseline may also be due to monocyte exhaustion (19, 20), with a moderate increase in monocyte %SRC following clearance of mycobacteria. However, the largest increase in %SRC of monocytes observed at 12 months may be explained by the lack of exposure to anti-TB drugs for approximately 6 months, as these TB drugs have been shown to modulate the bioenergetics of human macrophages (21).

Nineteen of the recruited TB patients had data at all the first three time points (M0, M2, and M6) during standard TB treatment. When the bioenergetic parameters of these TB cases during standard TB treatment were analyzed, the %SRC of lymphocytes showed significant longitudinal changes (Kruskal–Wallis statistic = 8.5296, *P* = 0.0111, **Supplementary Figure S3A**). Furthermore, assessment of the data of all the enrolled TB-positive cases (n = 38 at baseline) that did not have data at all four time points also revealed significant longitudinal changes in %SRC (Kruskal–Wallis statistic = 17.314, *P* = 0.0006, **Supplementary Figure S3B**) and Max Resp (Kruskal–Wallis statistic = 9.9130, *P* = 0.0193) of the lymphocytes (**Supplementary Figure S3C**), and in %SRC of the monocytes (Kruskal–Wallis statistic = 7.8244, *P* = 0.0498, **Supplementary Figure S3D**). At 12 months after initiating therapy, the %SRC of the lymphocytes of the TB patients returned to the levels observed in the lymphocytes of the controls (dotted line). However, at 12 months, the Max Resp of the lymphocytes and %SRC of the monocytes in TB patients did not return to the levels observed in the controls (dotted line).

These data suggest that the positive response to standard TB treatment increases the %SRC of circulating lymphocytes at M6 (end of treatment) and M12, and of monocytes at M12. Reprogramming of immune cells in cured TB patients is likely responsible for the increased %SRC and maximal respiration of immune cells at the end of treatment.

### Correlation of bioenergetic parameters with the clinical parameters

**Figure 5:**
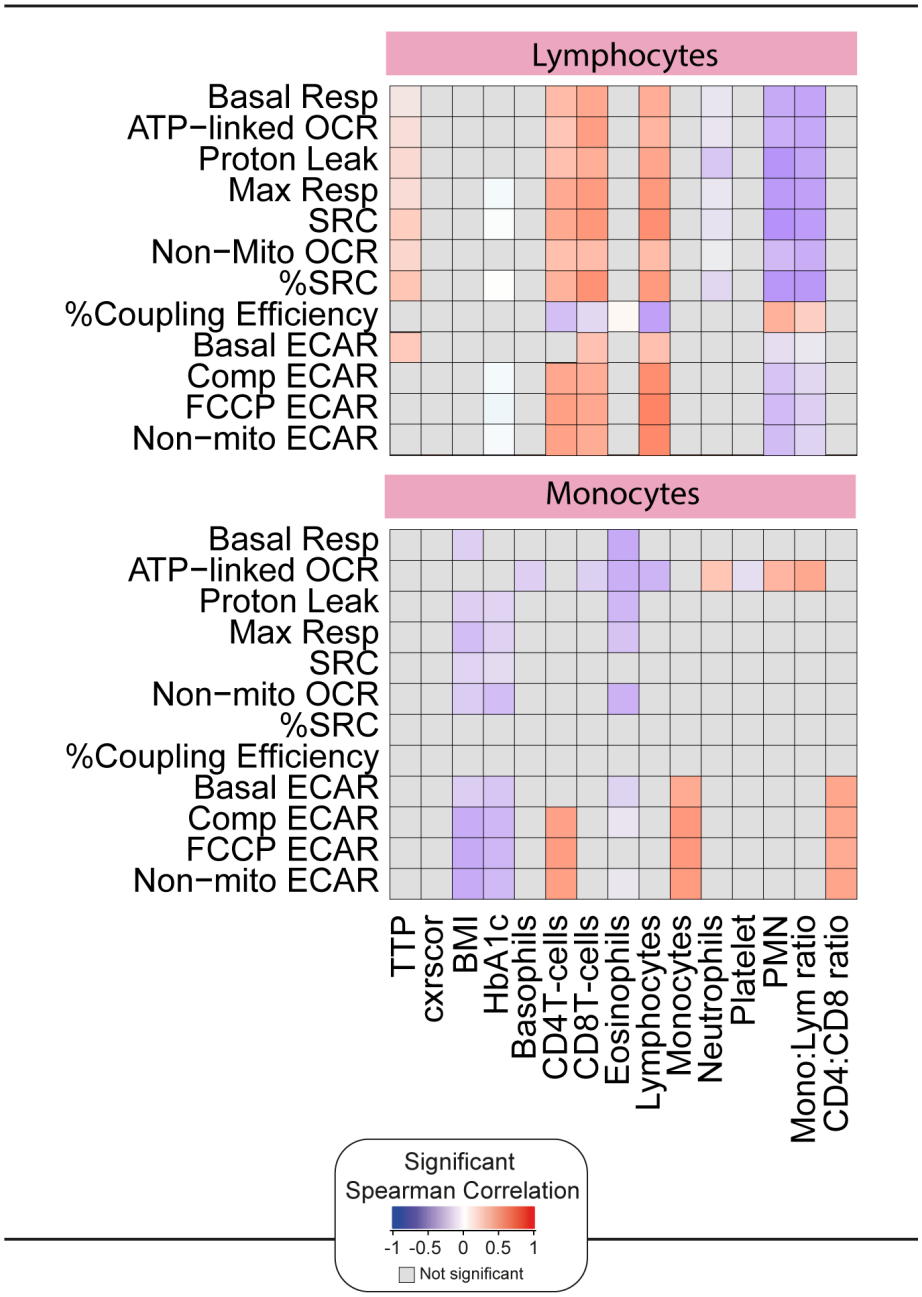
Correlation of the bioenergetic parameters with the clinical parameters. The heatmaps shows statistically significant correlations (p <0.05) between bioenergetic markers measured at baseline and clinical parameters. Each square represents a positive (red) or negative (blue) Spearman correlation, with the color varying according to correlation intensity. Gray square represents a not significant correlation. BMI, body mass index; cxrscor, chest X-Ray score; Mono, monocytes; Lym, lymphocytes; PMN, polymorphonuclear neutrophils; TTP, time to positivity.

To assess whether the impaired bioenergetics of the circulating immune cells of TB patients are associated with any clinical parameters at diagnosis, we performed a correlative analysis using Spearman’s rank test (**Figure 5**). Importantly, this was carried out prior to the initiation of TB treatment, and thus was not affected by the potential impact of TB drugs on some of the bioenergetic parameters (21). A significant correlation was observed between the bioenergetic parameters of lymphocytes and sputum bacterial load, as measured by time to positivity (TTP). Interestingly, this was not observed in monocytes. No correlations were observed between the bioenergetic parameters of either cell type and the extent of lung disease, as measured by X-ray (cxrscore).

Significant positive correlations were observed between the bioenergetic parameters of lymphocytes and the absolute numbers of CD4+ T cells, CD8+ T cells, and lymphocytes in whole blood. Exceptions included %Coupling Efficiency for all three cell types and basal ECAR for CD4+ T cells. Significant negative correlations were observed between the bioenergetic parameters of lymphocyte and neutrophil abundance and the monocyte:lymphocyte ratio. Both parameters are considered markers of inflammation, but it is possible that the observed differences are related to the relative reduction in lymphocyte number.

The ECAR bioenergetic parameters of monocytes were significantly correlated with the absolute numbers of monocytes, CD4+ T cells, and CD4:CD8 T cell ratio in whole blood. Interestingly, and distinct from lymphocytes, negative correlations were observed between the respiratory parameters of monocytes and body mass index (BMI) and HbA1c.

Taken together, these data suggest that, although highly downregulated at baseline, the *ex vivo* bioenergetic parameters measured by XF correlate with independent measures of TB disease and immune dysregulation. These associations were most prominent in lymphocytes. Therefore, we examined the association between lymphocyte parameters and plasma markers of inflammation measured by Luminex.

### Network analysis of the bioenergetic parameters of monocytes and lymphocytes

**Figure 6:**
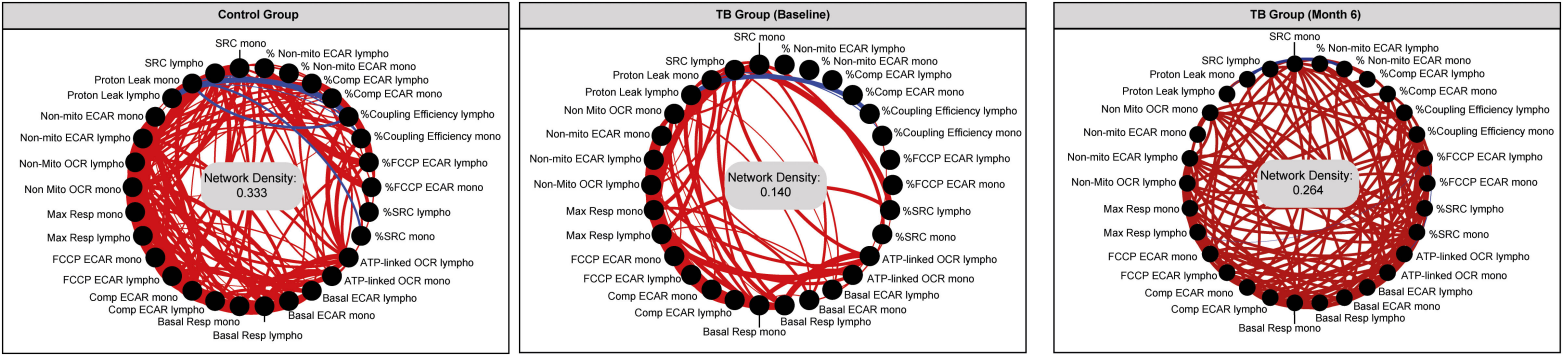
Network analysis of the bioenergetic parameters of the monocytes and lymphocytes of controls and TB patients reveals distinct interactions. The network analysis (interactome) shows statistically significant correlations, p <0.05 between all the bioenergetic parameters of the circulating monocytes (mono) and lymphocytes (lympho) measured in control and TB patients at baseline and month 6. Each node represents a biomarker, and edges represent strong Spearman correlations between markers, defined as |rho| >0.6. Blue lines indicate negative correlations and red lines indicate positive correlations. Network density was established calculating the known number of connections by potential connections. Mono, monocytes; Lympho, lymphocytes.

A statistical analysis known as a network analysis was then used to characterize the dynamics of the correlations between the values of the bioenergetic parameters of monocytes and lymphocytes over time (months 0 and 6) in TB patients and non-TB infected controls (**Figure 6**). Relative to the non-TB infected controls, there was reduced connectivity in TB patients at diagnosis (network density = 0.333 and 0.140, respectively), indicative of dysregulation in the bioenergetic metabolism of monocytes and lymphocytes in TB patients at diagnosis. However, at month 6 (M6), the network density of the bioenergetic parameters of the TB patients demonstrated increased connectivity (0.264), closer to that of the non-TB infected controls (0.333). These findings suggest that over time, the bioenergetic metabolism of immune cells in these individuals is improving and becoming more similar to the non-TB infected controls.

### Differential correlation of the clinical and bioenergetic parameters in the lymphocytes and monocytes

**Figure 7:**
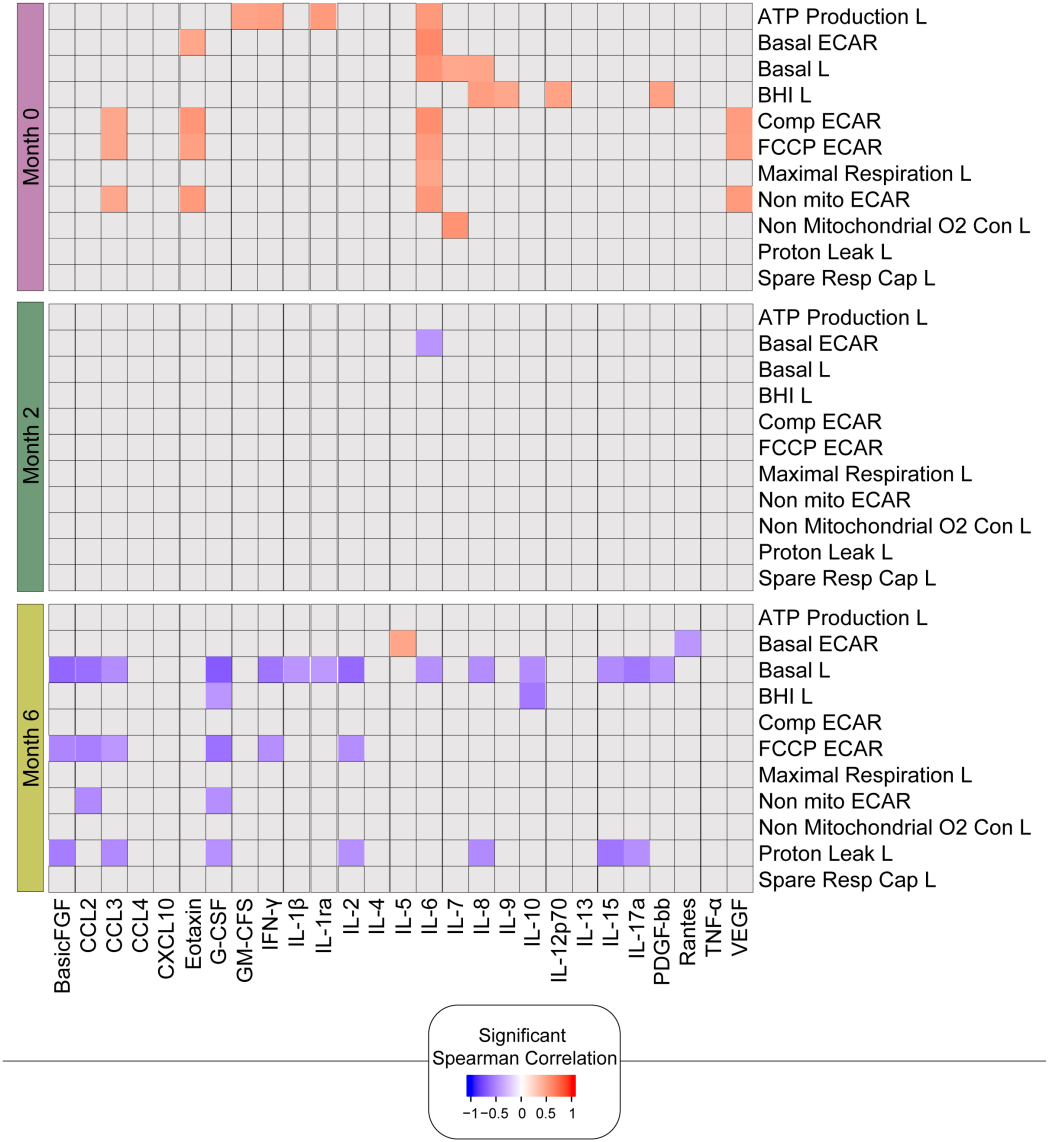
Bioenergetic markers are differently correlated with cytokines during anti-TB treatment. The heatmap shows statistically significant correlations (p <0.05) between bioenergetic and inflammatory markers measured across times. Each square represents a positive (red) or negative (blue) Spearman correlation, with the color varying according to correlation intensity. Gray square represents a non-significant correlation. L, lymphocyte.

As there were significant positive correlations between the bioenergetic parameters and the absolute numbers of lymphocytes (**Figure 5**), we next correlated the bioenergetic parameters of the lymphocytes with plasma cytokine levels (**Figure 7**). At baseline, significant positive correlations were observed between plasma levels of IL-6 and seven bioenergetic parameters of the lymphocytes of TB patients, including basal and maximal respiration, ATP-linked OCR, and basal and compensatory ECAR. Plasma levels of basic fibroblast factor (basic FGF) correlated positively with all four ECAR parameters (Basic, Comp, FCCP, and Non-mito), and vascular endothelial growth factor (VEGF) and CCL3 with three of the ECAR parameters (Comp, FCCP, and Non-mito). At month 2, the only remaining correlation was a negative correlation observed between basal ECAR and plasma levels of IL-6. At 6 months of treatment, at which time all participants were *Mtb* culture-negative, the significant correlations detected were primarily negative in the direction between basal respiration and several pro-inflammatory cytokines, including IL-17a, IL-1β, IL-6, INF-γ, and the chemokine CCL3.

In summary, these findings are consistent with the link between improved metabolic health and the resolution of inflammation following successful TB treatment. The changes in the correlations over time likely reflect a combination of the change in lymphocyte composition as well as the phenotype of these lymphocytes in response to clearance of the mycobacteria with a positive response to treatment.

### Network analysis shows that anti-TB treatment alters the bioenergetic parameters of circulating immune cells and the inflammatory profile

**Figure 8:**
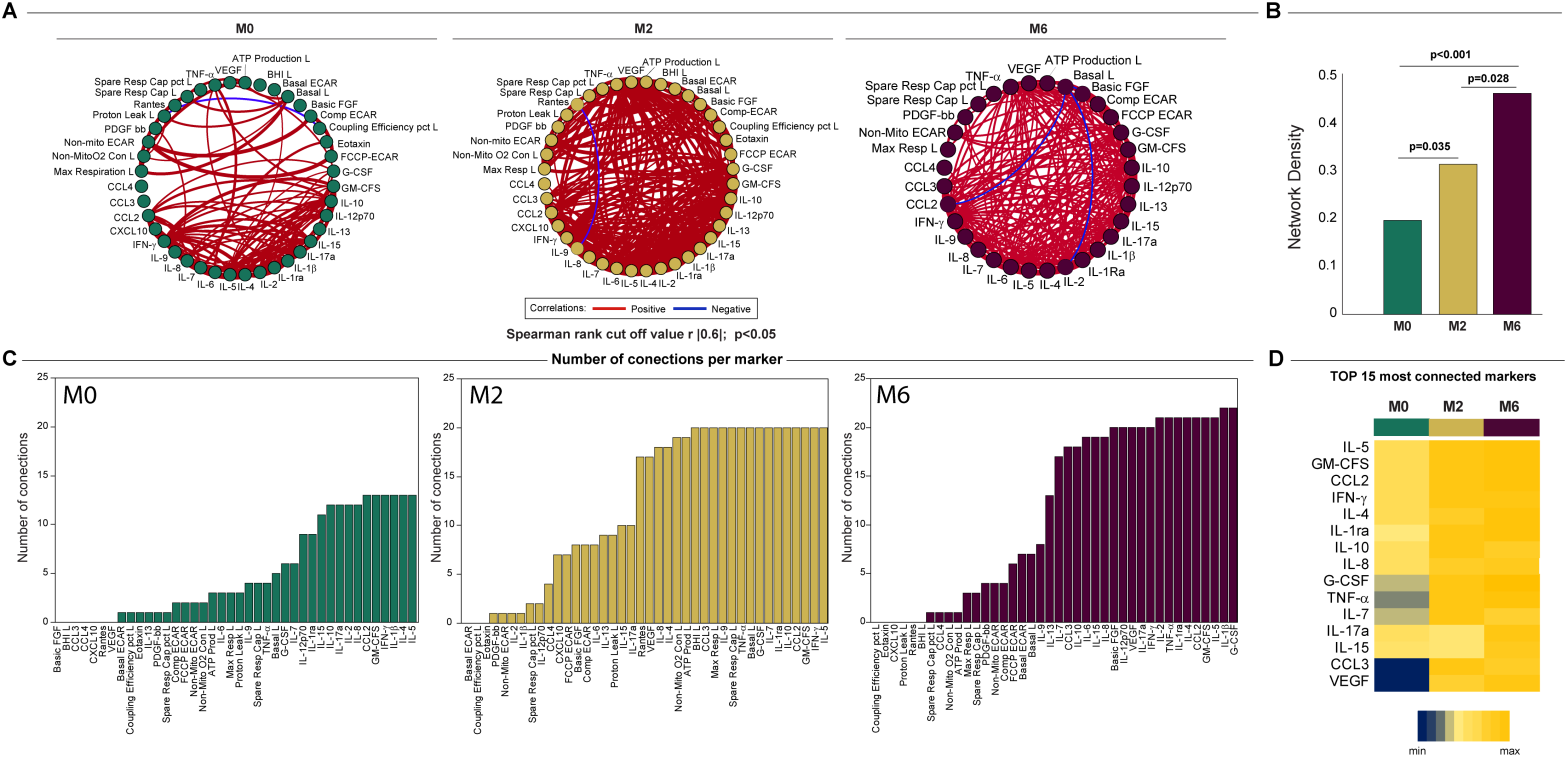
Network analysis of the inflammatory profile and bioenergetic parameters of lymphocytes reveals distinct changes induced by anti-TB treatment. **(A)** The network analysis (interactome) shows statistically significant correlations, p <0.05) between all the parameters measured across times. Each node represents a parameter, and edges represent strong correlations between parameters, defined as |rho| >0.6. **(B)** Data of network densities were compared between the indicated timepoints using the permutation test (56). **(C)** Histogram shows the parameters in descending order of the number of connections, for each time point, for the top 15 most connected markers. **(D)** Number of connections by parameter and timepoint. The scale variates from blue (lower number of connections) to yellow (higher number of connections). L, lymphocytes.

Network analysis was used to characterize the dynamics of the correlations between the values of the bioenergetic parameters of monocytes and lymphocytes and the serum cytokine levels over time in TB patients (months 0, 2, and 6). The network analysis demonstrated that the connectivity of the inflammatory profile and the bioenergetic parameters increased with treatment at month 2 (M2) and cure at month 6 (M6) in TB patients (**Figure 8A**), which indicates varying levels of coordination in the immune response. A more interconnected network (M6), with positive correlations suggests a highly coordinated response at this time point. This is highlighted by the comparison of the network densities at each time point in **Figure 8B**. The higher network density at M6 in comparison to that at baseline and M2, as well as the higher network density at M2 in comparison with that at baseline, indicated an increase in immune coordination through the time points (p <0.05). **Figure 8C** shows the highly connected parameters of M0, M2, and M6. **Figure 8D** indicates how the connectivity of each parameter changes across the time points, indicating its varying roles or importance at different time points. Notably, VEGF and CCL3 had a low number of connections at M0 but a higher number at M2 and M6, suggesting that the treatment can increase the levels and/or the involvement of this cytokine in the immune response. These findings suggest that the inflammatory profile and bioenergetic parameters increased with treatment, indicating varying levels of coordination in the immune response.

## Discussion

In this study, we investigated the bioenergetic parameters of monocytes and lymphocytes isolated from the blood of patients with culture-confirmed drug-susceptible TB at diagnosis and during standard TB treatment, to further our understanding of the role of bioenergetic metabolism in TB. Using extracellular flux analysis, we found a significant difference in the bioenergetic parameters between non-TB infected HIV-negative individuals and TB patients at diagnosis. The diminished bioenergetic parameters observed in monocytes and lymphocytes were found to align with key indicators of TB and immune dysregulation, particularly evident in measures such as sputum bacterial load and lymphocyte counts. Importantly, throughout treatment and post-treatment, there was a notable increase in the %SRC and Max Resp levels of lymphocytes, alongside a noticeable improvement in the %SRC of monocytes after 12 months. Consistent with this, network correlation analysis of bioenergetic parameters in both lymphocytes and monocytes revealed significantly fewer network connections upon TB diagnosis than in controls, consistent with immune metabolism dysregulation. Nonetheless, subsequent to effective drug therapy, the network connections increased to levels close to those of the control group. This trend persisted when plasma cytokines, measured at corresponding intervals, were factored into the correlation analysis, with baseline network connectivity being low, but increasing markedly throughout the course of treatment. These findings may help elucidate how bioenergetic changes impact immunity, lead to new immunomodulatory strategies in TB, and identify individuals at risk of disease progression or relapse.

SRC has been demonstrated to be critical in maintaining homeostasis in many cell types, with changes in SRC being associated with many non-communicable pathologies (22–31). Thus, the low %SRC observed in the monocytes and lymphocytes of TB patients at diagnosis is indicative of poor health of immune cells in people with TB. This may be due to exhaustion of the cells (32), as observed in a TB mouse model where CD8^+^ T cells, with a high expression of T-cell exhaustion markers, PD-1 and CTLA-4^+^, demonstrated significant reductions in SRC compared to healthy T cells (16). This is further supported by Day et al. (33) who observed upregulation in the inhibitory receptor PD-1 on naïve and memory CD4^+^ T cells isolated from the blood of TB patients with positive sputum smear microscopy. This is also a likely explanation for the reduced network density of the interactome between the bioenergetic parameters of the lymphocytes and monocytes isolated from TB patients at baseline in comparison to the network density of the interactions between the bioenergetic parameters of the cells isolated from TB uninfected HIV-negative controls.

However, in the early stages of TB infection, the reduced %SRC of monocytes and lymphocytes may also be indicative of an activated proinflammatory state of circulating immune cells. Pro-inflammatory macrophages enhance glycolysis and the pentose phosphate pathway (34, 35) while reducing OXPHOS and the associated %SRC to promote their functions of phagocytosis, ROS production, and cytokine secretion (36). Effector short-lived Th1, Th2, and Th17 lymphocytes also rely on glycolysis and glutaminolysis (37) to fuel inflammation and, to a lesser degree, on OXPHOS and, consequently, %SRC. Thus, the metabolism associated with the pro-inflammatory states of circulating immune cells might also contribute to the reduced %SRC at baseline.

The incremental changes in the %SRC with TB treatment at months 2 and 6 were likely due to a change in the composition of the circulating lymphocytes in addition to changes in the phenotype of the lymphocytes. CD8^+^ memory T cells isolated from wild-type mice infected with *Listeria monocytogenes* have been shown to have higher SRC than effector CD8^+^ T cells and naïve CD8^+^ T cells (29). As the absolute number and percentage of CD8^+^ T cells in TB patients at diagnosis correlated significantly with %SRC, it is possible that the increase in %SRC may be due to an increase in CD8^+^ memory T cells. Additionally, an increase in the number of circulating naïve T cells might lead to increases in SRC, as these are quiescent cells that have less mitochondrial mass and lower SRC than memory T cells (29, 38, 39).

When the bioenergetic parameters of the lymphocytes isolated from TB patients at diagnosis were correlated with the plasma levels of cytokines and chemokines, only IL-6 correlated positively with seven bioenergetic parameters of the lymphocytes (basal respiration, ATP-linked OCR, maximal respiration, and all the ECAR parameters) at baseline. After two months of therapy, other than basal ECAR, which correlated negatively with IL-6 levels, no correlations were observed between IL-6 and the bioenergetic parameters, suggesting resolution of inflammation. After 6 months of treatment, a positive correlation was only observed between levels of IL-6 and basal ECAR, suggesting that low plasma levels of IL-6 were associated with low basal ECAR. IL-6 displays pleiotropic activity but is promptly and transiently produced in response to infections and injuries (40). IL-6 has been associated with early protective responses in TB mediated by IFN-γ (41, 42), which is supported by significantly higher plasma levels of IL-6 in fast responders (sputum negative during the second month of anti-TB treatment) (43). Higher plasma concentrations of IL-6 have also been associated with a shorter time to culture conversion among HIV-infected individuals and were significantly associated with the presence of lung cavitation during active TB in a cohort from the Prince Zulu Communicable Disease Centre in Durban, South Africa (44). IL-6 plasma concentrations in TB patients have also been shown to decline during antimycobacterial treatment at 6 and 16 weeks (45), supporting the lack of a positive correlation between the plasma levels of IL-6 and the lymphocyte bioenergetic parameters at later time points during therapy. Basic fibroblast growth factor and VEGF, which demonstrated some positive correlations with lymphocyte bioenergetic parameters at baseline, are both angiogenic factors that act on endothelial cells (46). VEGF has been shown to be elevated in both the blood and sputum of pulmonary TB patients, with decreased levels observed with therapy (47, 48), which also verified the absence of correlations between VEGF and bioenergetic parameters at months 2 and 6. Notably, the spare respiratory capacity of lymphocytes did not correlate with any of the plasma levels of cytokines or chemokines.

The limitations of this study include the relatively small number of participants enrolled, of which not all were followed up at later time points, which limits the findings of this study to this population and associated baseline characteristics. In addition, the composition of the “lymphocyte” population includes all peripheral mononuclear cells minus monocytes, the composition of which may vary between participants and with treatment. Another limitation is the younger median age of the controls and the lower percentage of males recruited from the same district. Confounding factors affecting bioenergetic metabolism, such as smoking, malnutrition, and comorbidities such as atherosclerosis, cancer, and other opportunistic infections, may also impact the findings of this study (49–52). Further investigations of the metabolic pathways involved in the reprogramming of the immune cells and characterization of the lymphocyte population were prohibited by the small blood sample volume drawn from the participants, as TB infection is often associated with neutropenia, anemia, and thrombocytopenia (53).

In conclusion, this study demonstrated that the bioenergetic metabolism of circulating lymphocytes and monocytes in TB patients at diagnosis is suppressed when compared to healthy controls from the same study site. Furthermore, there is a longitudinal increase in bioenergetic metabolism, specifically %SRC, of the circulating lymphocyte population with a positive response to TB treatment. Longitudinal changes from positive to negative correlations between ECAR parameters and cytokine responses suggest reduced inflammation in response to mycobacterial clearance. However, further research is required to understand the mechanisms underlying these bioenergetic changes. Further research is needed using tissue-specific analyses to explore metabolic reprogramming within TB granulomas or other infected tissues to provide additional insights. Here, we have generated new knowledge that may help elucidate how bioenergetic changes impact immunity, which underscores the need for further research on new immunomodulatory strategies and identify individuals at risk of disease progression or relapse.

These findings have important implications for examining how bioenergetic changes affect immunity, which may lead to new immunomodulatory strategies and help identify individuals at risk of disease progression or relapse.

## Materials and methods

### Study population

Participants were enrolled from the KwaDabeka Clinic in KwaZulu-Natal, Durban, as part of the Regional Prospective Observational Research for Tuberculosis (RePORT), South Africa study, between December 2016 and December 2019 (54). All participants were at least 18 years old, displayed symptoms of TB, were GeneXpert and culture-positive (in solid and/or liquid media), had chest X-ray evidence of pulmonary involvement, and provided written informed consent. All RePORT study participants were followed up for the duration of standard 6-month anti-TB treatment for drug-susceptible TB up to 12 months after enrolment. Participants who were resistant to any of the standard anti-TB drugs (isoniazid, rifampicin, ethambutol, or pyrazinamide) at any time during the study were excluded from the study. A cure was defined as a negative sputum culture result at the end of treatment, which was based on the completion of the standard therapy for drug-susceptible TB with no evidence of failure.

### Controls

A group of healthy, asymptomatic, HIV-negative individuals was recruited from the Collection of Urine, Blood and Sputum (CUBS) cohort from the same community where TB patients were recruited.

### Descriptive statistics

Age, sex, race, body mass index (BMI), self-reported alcohol use, tobacco use, employment, and HIV status were recorded. For HIV-infected participants, the date of commencement of antiretroviral (ARV) therapy, CD4^+^ T cell, and CD8^+^ T cell absolute counts were measured. Hemoglobin A1c (HbA1c) levels were collected from all TB participants and healthy controls at enrolment. The bacterial burden was estimated from the “time to positivity” of the liquid culture of the sputum using the BD BACTEC MGIT 960 mycobacterial detection system (Becton Dickinson Microbiology Systems, Sparks, MD, USA). CD4^+^ and CD8^+^ T cell counts were measured in an independent commercial laboratory. For healthy controls, age, sex, and race were recorded, and HbA1c levels were measured.

### Sample collection, processing, and extracellular flux analysis

Blood and sputum samples were collected from culture-confirmed TB participants at baseline (enrolment), M2, month M6, and M12 post-enrolment. Blood samples were collected from the ACD vacutainers (BD Biosciences). Peripheral blood mononuclear cells (PBMCs) were isolated from whole blood using Sepmate™ Tubes. Briefly, 15 ml Histopaque^®^ 1077 was pipetted through the central hole in the insert into the tubes. Blood was diluted to an equal volume in Dulbecco’s phosphate-buffered saline (DPBS) with 2% FBS and pipetted down the side of the upright Sepmate™ tube. The samples were centrifuged at 1,200×*g* for 10 min at room temperature with a brake on. PBMC were harvested by pouring the top layer into a new tube in a smooth motion. PBMC were washed twice with DPBS (50 ml, 400×*g*, 10 min) and resuspended in 5 ml cold separation buffer (DPBS, 2 mM EDTA, 0.5% (w/v) BSA).

CD14^+^ monocytes were isolated by magnetic cell sorting using MACS CD14-microbeads (Miltenyi, 130-505-201) and the flow through was collected as “lymphocytes.” The cells were washed with DPBS, counted, and resuspended in CMST medium (DMEM, 30 mM NaCl, 5 mM HEPES, 2 mM L-Glutamax, 1 mM sodium pyruvate, pH 7.4, 37°C) to a final cell concentration of 1.875 × 10^6^ cells/mL. The wells of the XFp microtiter plate were coated with Cell-Tak™ (Corning) as follows: 60 µl dH_2_O and 180 µl 0.1 M NaHCO_3_ (pH 8.0) were added to 30 µl Cell Tak™, the pH was adjusted to 7.2–7.8, and 10 µl of the solution was added to each well of the XF*P* cell microtiter plate. After incubation at room temperature for 20 min, each well was washed twice with 100 μl of sterile water and left to dry under sterile conditions. Cells were seeded into XFp cell culture plates at a density of 150,000 cells per well in a volume of 80 µl CMST media, and the cells were pelleted in the wells coated with Cell-Tak™ by centrifugation at 800×*g* for 1 min with a break in the lowest setting. The orientation of the plate was changed, and the cells were centrifuged at 1,100×*g* for 1 min with a break in the lowest setting. The final volume was brought up to 180 µl with CMST medium and the medium in the cell plate was degassed for a minimum of 30 min in a non-CO_2_ incubator.

The mitochondrial modulators oligomycin, carbonyl cyanide-4 (trifluoromethoxy) phenylhydrazone (FCCP), rotenone, and antimycin A were prepared in CMST medium from DMSO stocks at 10× the concentrations listed in **Table 2**. The pH of the solutions was adjusted to 7.4 at 37°C and loaded into the ports of the XFp cartridge as previously described (15). The extracellular flux of the cells was analyzed using the Cell Mito Stress Test protocol on an XFp with 3 min of mixing and 4-minute measurements. CMST was performed within 8 h of blood collection from the participants in the study.

**Table 2:** Concentrations of respiration modulators used in the CMST assay.

	Concentration
Oligomycin	0.5 µM
FCCP	0.6 µM
Rotenone and Antimycin	2.0 µM

### Normalization of extracellular flux by protein concentration

Following XF analysis, the media from all wells were aspirated, leaving 10 µl, and the cells were fixed with 10 µl formalin. The cells in each well were lysed using 20 µl of 25 mM NaOH. Bradford reagent (150 µl, BioRad 500-0205) was added to each well and the plate was incubated in the dark for 5 min. A protein standard curve was set up in another XFp cell microtiter plate using BSA standards (5 µl) ranging from 0.125 mg/ml to 2 mg/ml (BioRad 500-0202) and treated with formalin, NaOH, and Bradford reagent, as described for the cells. The absorbance of each well was measured at 595 nm wavelength using a Biotek Synergy H4 Hybrid spectrophotometer. The CMST assay parameters were calculated using the CMST Report Generator on Agilent Seahorse Wave desktop software (version 2.6). Respiratory parameter data (OCR and ECAR) were normalized using protein concentration and exported using the Agilent Seahorse Biosciences Cell Mito Stress Test report generator for further analysis.

### Cytokine analysis

Plasma samples from the participants were purified and stored at −80°C prior to batch-wise Luminex assays (Bio-Rad, Hercules, CA) using the Bio-Plex Human Cytokine 27-plex assay (Bio-Rad, Hercules, CA, USA), as previously described (55). This panel examined the levels of FGF basic, Eotaxin, G-CSF, GM-CSF, IFN-γ, IL-1β, IL-1ra, IL-2, IL-4, IL-5, IL-6, IL-7, IL-8, IL-9, IL-10, IL-12 (p70), IL-13, IL-15, IL-17A, IP-10, MCP-1 (MCAF), MIP-1α, MIP-1β, PDGF-BB, RANTES, TNF-α, and VEGF.

### Spearman correlation and network analysis

We conducted Spearman’s correlation analyses among inflammatory, clinical, and bioenergetic parameters. For this, we used the Spearman rank test |rho| and considered significant correlations with a p-value of less than 0.05. The inferential networks were generated from Spearman correlation matrices containing the values of the bioenergetic parameters of lymphocytes and plasma cytokine levels. The |rho|. was used to assess strong correlations between all biomarkers at each time point, where correlations were considered significant if |rho| ≥0.60 and p <0.05. Correlation networks were constructed using Gephi software (https://gephi.org/). To analyze the structure of the networks, network density was calculated, which is the ratio of the number of edges inferred in the network to the total number of possible edges between all pairs of nodes. The density measure is defined as follows: density = L/(N [N − 1]/2), where L is the number of observed edges (Spearman correlations with |rho| >0.6 and p <0.05) and N is the total number of nodes in the network. The density was normalized, ranging from 0 (no edges in the network) to 1 (all possible edges present). The number of statistically significant correlations between the time points was compared using a permutation test. Graphics for network analysis were customized using JMP Pro 13 (JMP Statistical Discovery LLC) and Adobe Illustrator (Adobe Systems Inc.).

### Statistical analysis

Baseline characteristics were compared between particiapants with drug-susceptible culture-confirmed TB and healthy controls using Chi-square or Fisher’s exact test for categorical variables and Wilcoxon rank sum and Kruskal–Wallis test for continuous variables. Differences in distributions and medians of the bioenergetic parameters were compared between culture-confirmed TB participants and healthy controls at baseline, and changes in culture-confirmed participants from baseline during TB treatment were examined using the Wilcoxon rank-sum and Kruskal–Wallis tests.

Spearman’s correlation analyses were done to examine associations between the CD4^+^ T cell counts, CD8^+^ T cell counts and %SRC and Maximal respiration, in addition to associations between the bioenergetic parameters and the cytokine levels. Statistical analysis was performed using SAS for Windows version 8.3 (SAS Institute, Cary, NC, USA) and GraphPad PRISM, version 9.1.1 (GraphPad Software, San Diego, CA, USA).

## Data Availability

The raw data supporting the conclusions of this article will be made available by the authors, without undue reservation.
