# Corrigendum: Longitudinal mitochondrial bioenergetic signatures of blood monocytes and lymphocytes improve during treatment of drug-susceptible pulmonary tuberculosis patients

**DOI:** 10.3389/fimmu.2025.1557327

**Published:** 2025-01-30

**Authors:** Bridgette M. Cumming, Kelvin W. Addicott, Fernanda Maruri, Vanessa Pillay, Rukaya Asmal, Sashen Moodley, Beatriz Barreto-Durate, Mariana Araújo-Pereira, Matilda Mazibuko, Zoey Mhlane, Nikiwe Mbatha, Khadija Khan, Senamile Makhari, Farina Karim, Lauren Peetluk, Alexander S. Pym, Mahomed Yunus S. Moosa, Yuri F. van der Heijden, Timothy S. Sterling, Bruno B. Andrade, Alasdair Leslie, Adrie J. C. Steyn

**Affiliations:** ^1^ Africa Health Research Institute, University of KwaZulu-Natal, Durban, South Africa; ^2^ Vanderbilt Tuberculosis Center, Vanderbilt University School of Medicine, Nashville, TN, United States; ^3^ Division of Infectious Diseases, Department of Medicine, Vanderbilt University School of Medicine, Nashville, TN, United States; ^4^ Multinational Organization Network Sponsoring Translational and Epidemiological Research (MONSTER) Initiative, Salvador, Brazil; ^5^ Laboratório de Pesquisa Clínica e Translacional, Instituto Gonçalo Moniz, Fundação Oswaldo Cruz, Salvador, Bahia, Brazil; ^6^ Department of Infectious Diseases, University of KwaZulu-Natal, Durban, South Africa; ^7^ Global Division, The Aurum Institute, Johannesburg, South Africa; ^8^ Department of Infection and Immunity, University College of London, London, United Kingdom; ^9^ Department of Microbiology, University of Alabama at Birmingham, Birmingham, AL, United States; ^10^ Centers for AIDS Research and Free Radical Biology, University of Alabama at Birmingham, Birmingham, AL, United States

**Keywords:** tuberculosis, bioenergetic metabolism, lymphocytes, monocytes, TB treatment, cytokines, Seahorse XF96

In the published article, there was an error in the article title. Instead of “Longitudinal mitochondrial bioenergetic signatures of blood monocytes and lymphocytes improve during treatment of drug-susceptible pulmonary tuberculosis patients Monocyte/lymphocyte bioenergetic signatures post-TB treatment”, it should be “Longitudinal mitochondrial bioenergetic signatures of blood monocytes and lymphocytes improve during treatment of drug-susceptible pulmonary tuberculosis patients”.

The authors apologize for this error and state that this does not change the scientific conclusions of the article in any way. The original article has been updated.

