# Supplementary material for: Longitudinal mitochondrial bioenergetic signatures of blood monocytes and lymphocytes improve during treatment of drug-susceptible pulmonary tuberculosis patients
Source: Front Immunol. 2024 Nov 13;15:1465448. doi: 10.3389/fimmu.2024.1465448 (PMC11599235; doi:10.3389/fimmu.2024.1465448)
Supplement: Supplementary file 1 [file DataSheet1.docx]

**Supplementary Figures**

**A**


 **Figure S1:** Effect of HIV status of TB patients on the bioenergetic parameters of the (**A**) lymphocytes, and (**B**) monocytes. Comp, compensatory.

**B**

**A**

**Figure S2:** Effect of HbA1c levels on the bioenergetic parameters of the (**A**) lymphocytes, (**B**) monocytes.

**Figure S3****:** **Longitudinal assessment of %SRC and maximal respiration of lymphocytes and monocytes of TB patients during and after standard treatment.**

(**A**) Changes in %SRC of lymphocytes of TB patients with data at all first three timepoints (n = 19); (**B-D**) Changes in (**B**) %SRC and (**C**) maximal respiration of lymphocytes, and (**D**) %SRC of the monocytes of all TB participants (GeneXpert positive, solid culture positive) recruited and analyzed at diagnosis and at one or more follow-up timepoints. Dotted lines represent the median control levels. KW, Kruskal-Wallis.
